# CT Analysis of Variations in the Medial Maxillary Wall Relative to the Medial Orbital Wall: Implications for Surgical Risk Stratification from an Endoscopic Perspective

**DOI:** 10.3390/life15030453

**Published:** 2025-03-13

**Authors:** Humaid Alhumaid, Abdulrahman Alsowinea, Ali Alamer

**Affiliations:** 1 Department of Otolaryngology Head and Neck Surgery, College of Medicine, Qassim University, Buraydah 52571, Saudi Arabia; 3655@qu.edu.sa; 2 College of Medicine, Qassim University, Buraydah 52571, Saudi Arabia; a.f.sowinea@outlook.com; 3 Department of Radiology, College of Medicine, Qassim University, Buraydah 52571, Saudi Arabia

**Keywords:** computed tomography, maxillary sinus, lamina papyracea, medial orbital wall, inferomedial orbital strut, endoscopic sinus surgery

## Abstract

Functional endoscopic sinus Surgery (FESS) is a form of safe and effective management for chronic rhinosinusitis. Nevertheless, although FESS is minimally invasive, it poses a risk of rare orbital complications. This study aims to investigate the variations in the medial maxillary wall relative to the medial orbital wall, as depicted on computed tomography (CT) scans. We retrospectively included CT scans of the sinuses between November 2022 and April 2023. To maintain consistency, we used the coronal image that delineated the anterior ethmoidal foramen. The attachment site of the inferior turbinate to the medial maxillary wall was categorized into three classes according to its position relative to the inferomedial orbital strut. Class I indicates that the site of attachment is located within 2 mm, either medially or laterally. Class II indicates that it has been medially displaced by more than 2 mm, whereas Class III indicates that it has been laterally displaced by more than 2 mm. We enrolled 183 patients, yielding a total of 363 sides. Classes I, II, and III account for 55.4%, 41.3%, and 3.3% of the cases, respectively. A significant correlation exists between the classification and the dimensions and volume of the maxillary sinus (*p* < 0.001). The logistic regression model indicates a significant negative correlation between the width of the maxillary sinus and risk classification (*p* < 0.001), implying a protective effect with increasing width. Knowledge of the variations in the medial wall of the maxillary sinus relative to the medial orbital wall is essential for guidance toward the optimal endoscopic approach, and it demonstrates relevance to risk stratification.

## 1. Introduction

Surgical procedures for treating recurrent or refractory sinusitis have evolved throughout the years from invasive external approaches to minimally invasive endoscopic techniques [1]. Functional endoscopic sinus surgery (FESS) has become the preferred surgical approach for managing sinonasal obstruction, with the goal of improving mucociliary clearance by widening sinus ostia and restoring adequate sinus aeration [2,3]. The number of endoscopic sinus surgeries has rapidly escalated, and the procedure has expanded to include conditions beyond sinusitis. It is estimated that more than 250,000 patients undergo sinus surgery each year in the United States alone, with a minimal complication rate, affirming the safety of these procedures [4]. The clinical outcomes of FESS are well documented, with over 75% of patients reporting postoperative improvement in symptoms and quality of life [5].

However, despite being minimally invasive, FESS carries the potential for complications that are rarely severe, life-threatening, or even fatal. Cerebrospinal fluid leakage, meningitis, hemorrhage, and orbital injury are among the serious complications that have been reported [6,7]. Krings et al. analyzed the data of 78,944 patients who underwent FESS and found an overall major complication rate of 0.36%, which included orbital complications at 0.23% [8]. The orbital complications include injury to the lamina papyracea (LP), lacrimal duct injury, retro-orbital hematoma, optic nerve injury, and extraocular muscle injury [9]. The anatomical proximity of the orbit to the paranasal sinuses, along with the thin bone structures of the medial orbital wall, increases the risk of injury to the orbit during FESS [10]. Moreover, the LP, the main structure of the medial orbital wall, is susceptible to anatomical variations, which may be considered a potential cause of orbital complications [11]. Deniz et al. observed LP dehiscence in 2% of their cases; 60% of those had herniation of orbital contents into the ethmoid sinus, including orbital adipose tissue (83.3%) and medial rectus muscle (16.7%) [12]. Herniation of orbital adipose tissue was shown to be most prevalent in and around the anterior ethmoidal foramen, which is an important landmark in FESS, as traction on the anterior ethmoidal artery can result in devastating hemorrhage [13]. Another important surgical landmark relevant to the medial orbital wall is the uncinate process. Forceful manipulation of the uncinate process during FESS may result in the disruption of the medial orbital wall, especially as 52% of its superior attachment is to the LP [10,14].

Inadvertent injury to the LP often occurs in its anterior part during uncinectomy, middle meatal antrostomy (MMA), or while resecting the ethmoid bulla [15]. Therefore, recognizing the position and orientation of the LP with respect to the medial maxillary wall during preoperative planning might mitigate the risk of injury to the LP and subsequent violation of the orbital contents. The medial orbital wall typically aligns vertically with the maxillary ostium. However, in 10% of cases, the medial orbital wall is located medially to the maxillary ostium due to ethmoid hypoplasia, as observed in a previous study [16]. In such cases, the LP may be incorrectly recognized as an ethmoid sinus septation during surgery and predispose the patient to orbital injury. Herzallah et al. analyze the LP’s position on computed tomography (CT) and categorize it into three types based on a line traversing the attachment site of the inferior turbinate to the lateral nasal wall, which corresponds to the inferior margin of the MMA [17]. The Type I LP is located within 2 mm on both sides of this line. The Type II LP is located medially to this line by more than 2 mm. The Type III LP is located laterally to this line by more than 2 mm. El-Anwar et al. analyzed the position of the LP in patients with nasal polyps and discovered that those with larger polyps (grade III or IV) exhibited a significantly more medial LP compared to those with smaller polyps [18]. This suggests that the position of the LP may be influenced by various pathologies. Açar et al. examined the correlation between morphometric measurements of the orbit and the types of LP, discovering that orbital volume was greater in Type I [19].

In this study, we aim to analyze the positional variations in the medial maxillary wall relative to the medial orbital wall, as observed in CT scans. We used the anterior ethmoidal foramen and the inferomedial orbital strut (IOS) as landmarks to ensure consistency across patients. By analyzing these variations, we seek to develop a modified classification system that emphasizes the potential risks associated with surgical interventions in this region. We correlate this classification with maxillary sinus dimensions and volume. We also examined the impact of maxillary opacification, ostiomeatal obstruction, and other variations such as concha bullosa, paradoxical middle turbinate, and Haller’s cells. Understanding these variations will not only augment our knowledge of radiological anatomy but also serve as a practical tool for surgeons in preoperative planning, ultimately enhancing patient safety and efficacy in FESS. 

## 2. Materials and Methods

### 2.1. Study Setting and Participants

The Committee of Research Ethics at the Deanship of Scientific Research, Qassim University, Saudi Arabia, granted ethical approval for this study, No. 23-31-06, on 27 March 2023. We retrospectively included all patients who underwent CT scans of the paranasal sinuses at our institution between November 2022 and April 2023. These scans were performed as part of clinical evaluations for symptoms related to the sinonasal region (e.g., sinonasal obstruction) or for routine preoperative planning for endoscopic sinus surgery. Exclusion criteria included the following: (1) patients younger than 18 years; (2) those with extensive opacification of the sinonasal cavity causing bony rarefaction and hindering a proper assessment of the inferior margin of the MMA; (3) a history of prior sinus surgery; and (4) suboptimal CT image quality. We accessed the data between 24 December 2023 and 22 April 2024.

### 2.2. CT Scanning Techniques for the Paranasal Sinuses

All patients underwent a CT scan of the paranasal sinuses using the GE Revolution EVO 128-slice scanner without intravenous contrast media. The source axial CT scan images were acquired with the following parameters: a slice thickness of 0.625 mm, a tube voltage of 120 kilovolts (kV), and a tube current between 80 and 160 milliamperes (mA). Furthermore, multiplanar reconstruction (MPR) was employed to provide coronal and sagittal images of the paranasal sinuses. The Philips Vue Picture Archiving and Communication System (PACS) (©2022 Koninklijke Philips N.V.) was utilized for the purpose of image viewing. The right and left sides were graded independently. Consensus labelling was achieved by a neuroradiologist and an otolaryngologist, both with more than ten years of experience in their specialties.

### 2.3. Interpretation of Paranasal Sinus CT Scan Images

#### 2.3.1. Variations in the Medial Wall of the Maxillary Sinus Relative to the Medial Orbital Wall

**Figure 1:**
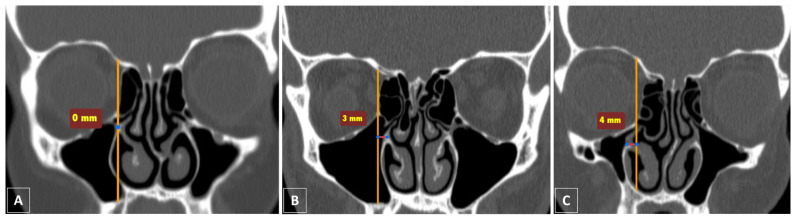
Classification of variations in the medial wall of the maxillary sinus relative to the medial orbital wall, as observed in coronal CT scan images of the paranasal sinuses. (**A**) Class I: the attachment site of the inferior turbinate is positioned in the same vertical plane as the line that crosses the IOS. (**B**) Class II: the attachment site of the inferior turbinate is positioned 3 mm medially to the vertical line. (**C**) Class III: the attachment site of the inferior turbinate is positioned 4 mm laterally to the vertical line.

Using the coronal CT scan images on both sides, we analyzed positional variations in the medial wall of the maxillary sinus (lateral nasal wall) relative to the medial orbital wall (formed primarily by the LP). In order to ensure consistency across all patients, the coronal image that clearly identified the location of the anterior ethmoidal foramen was used. A true vertical line was then drawn using the Annotations and Measurements tools in the Vue PACS, intersecting the junction between the medial orbital wall and the inferior orbital wall (IOS). The attachment site of the inferior turbinate to the medial wall of the maxillary sinus, which forms the inferior margin of the MMA, was categorized into three classes relative to this line. Class I is defined as any case in which the attachment site of the inferior turbinate is positioned within a 2 mm distance, either medially or laterally, from the vertical line. Class II is defined as any case in which the attachment site of the inferior turbinate is positioned medially more than 2 mm from the vertical line. Class III is defined as any case in which the attachment site of the inferior turbinate is positioned laterally more than 2 mm from the vertical line. This classification was modified from the earlier published classification by Herzallah et al. [17]. Figure 1 depicts the three classes in the coronal CT scan images.

#### 2.3.2. Dimensions and Volume of the Maxillary Sinus

We measured the height, width, and anteroposterior (AP) dimensions of the maxillary sinus on both sides. The height of the maxillary sinus was measured using the coronal CT image, and it was determined as the maximum distance between the floor and roof of the sinus. The width of the maxillary sinus was measured on the coronal CT image and determined as the maximum distance between the medial and lateral walls of the sinus. The AP dimension was measured on the sagittal CT image and determined as the maximum distance between the anterior and posterior walls of the sinus. The volume (cm^3^) of the maxillary sinus was determined using the formula (height × width × AP × 0.52) [20]. 

#### 2.3.3. Grading of the Opacifications in the Maxillary Sinus and Ostiomeatal Complex (OMC)

The opacifications in the maxillary sinus and OMC were assessed using the Lund–Mackay score and modified Lund–Mackay (Zinreich) score on both sides [21,22]. The maxillary sinus was categorized as no abnormality (complete air-filled sinus), partial opacification, or complete opacification. The OMC was categorized as no abnormality, partially obstructed, or completely obstructed. 

#### 2.3.4. Associated Variations

All cases were evaluated for the presence of concha bullosa (middle turbinate pneumatization), paradoxical middle turbinate (inferomedially curved middle turbinate), and Haller’s cells (infraorbital ethmoid air cells) on both sides. 

### 2.4. Statistical Analysis 

The frequencies and percentages of categorical variables were determined, whereas measures such as mean, median, and standard deviation (SD) were utilized to assess continuous variables. This study employed logistic regression to investigate the correlation between the dependent and independent variables. For statistical comparisons, the chi-square test, *t*-test, and one-way analysis of variance (ANOVA) were applied. A significance level of *p* < 0.05 was established.

## 3. Results

### 3.1. Demographic Data

A total of 231 CT scans of the paranasal sinuses were retrieved from the PACS. Out of the total number of cases, 48 (20.8%) were excluded for the following reasons: an age less than 18 years (n = 22, 45.8%), complete sinonasal opacification (n = 12, 25.0%), prior surgery (n = 10, 20.8%), and suboptimal CT techniques (n = 4, 8.3%). The study included the remaining 183 cases, which accounted for 79.2% of the total. The mean age of the patients was 30 years, with an SD of 11, while the median age was 26 years. Table 1 provides an overview of the frequencies of the participants’ ages. Males comprised 54.1% (n = 99), and females comprised 45.9% (n = 84). No statistically significant difference in gender was identified within our study cohort (*p* = 0.270). We analyzed the right and left sides as our unit of analysis, resulting in a total of 366 cases. Three cases were later excluded because of unilateral complete sinonasal opacification.

### 3.2. Dimensions and Volume of the Maxillary Sinus

The mean dimensions of the maxillary sinus in our sample were 35.6 mm (SD 5.91) for height, 26.0 mm (SD 4.45) for width, and 35.2 mm (SD 3.9) for the AP dimension. The mean volume was 17.4 cm^3^, with an SD of 6.1 cm^3^. Statistical significance was observed in the dimensions and volume of the maxillary sinus when analyzed across various age groups (Table 1), except for the AP dimension, which exhibited marginal significance (*p* = 0.052). A statistically significant difference was observed in maxillary sinus dimensions and volume when correlated with gender but not when correlated with laterality (Table 2).

### 3.3. Variations in the Medial Wall of the Maxillary Sinus Relative to the Medial Orbital Wall

**Figure 2:**
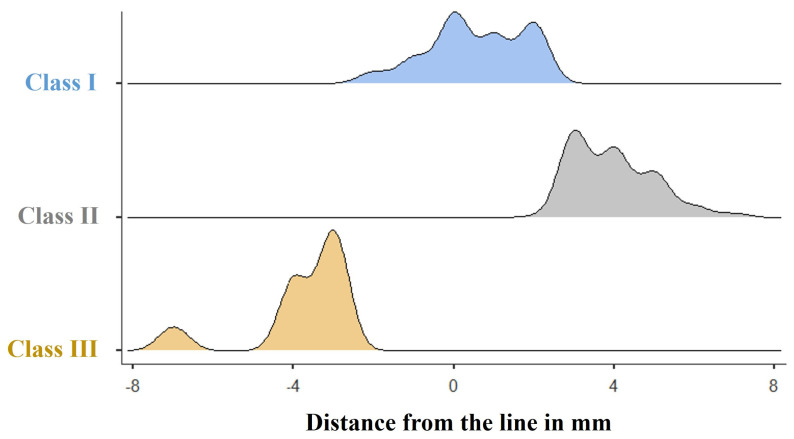
Density plots displaying the distance, in mm, between the proposed vertical line and the site of attachment of the inferior turbinate (medial maxillary wall) among the three classes.

According to the proposed vertical line that intersects the IOS, most cases (n = 201, 55.4%) were classified as Class I. Furthermore, 41.3% of cases (n = 150) exhibited a medial displacement of the medial maxillary wall and were classified as Class II. Finally, only 3.3% (n = 12) of cases exhibited a lateral displacement of the medial maxillary wall and were classified as Class III. The average distance from the attachment site of the inferior turbinate to the vertical line was 1.0 mm (either medially or laterally), 4 mm (medially), and 3.6 mm (laterally) in Classes I, II, and III, respectively (Figure 2). Table 3 illustrates the categorization of the cases into three classes and their correlation with laterality (right and left), average distance from the proposed vertical line, and gender. No statistically significant differences were observed among the three classes for age, gender, or laterality within our cohort.

### 3.4. Correlation of the Classification of the Medial Maxillary Wall with Maxillary Sinus Dimensions and Volume

**Figure 3:**
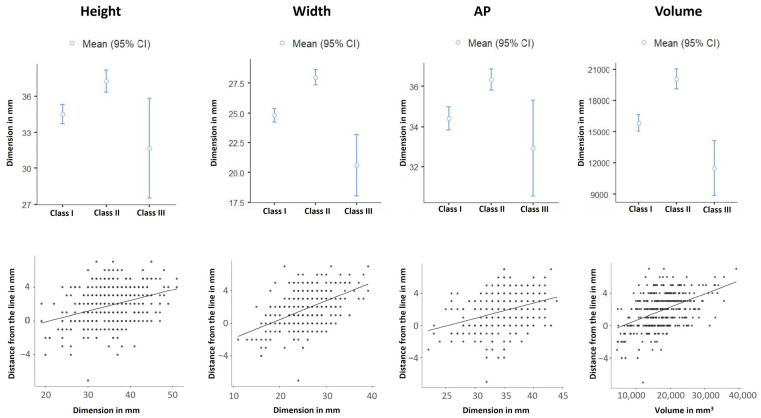
Box plots (upper row) and scatter plots with mean lines (lower row) displaying the correlation of the classification of the medial maxillary wall with maxillary sinus dimensions and volume in addition to distance from the vertical line in mm.

A statistically significant correlation was observed between the proposed classification and all maxillary sinus dimensions (height, width, and AP) as well as volume (*p* < 0.001). Table 4 and Figure 3 display the three classes together with the means and SDs of maxillary sinus dimensions and volume. 

### 3.5. Correlation of the Classification of the Medial Maxillary Wall with Maxillary Sinus Opacifications, OMC, and Associated Variations

The modified Lund–Mackay (Zinreich) score of the maxillary sinus and the OMC do not statistically significantly correlate with the proposed classification of the medial maxillary wall (Table 5). A statistically significant correlation exists between the proposed classification and Haller’s cells (*p* = 0.030). Nonetheless, there is no statistically significant association observed with concha bullosa or paradoxical middle turbinate. 

### 3.6. Correlation of the Classification of the Medial Maxillary Wall with Risk Stratifications 

**Figure 4:**
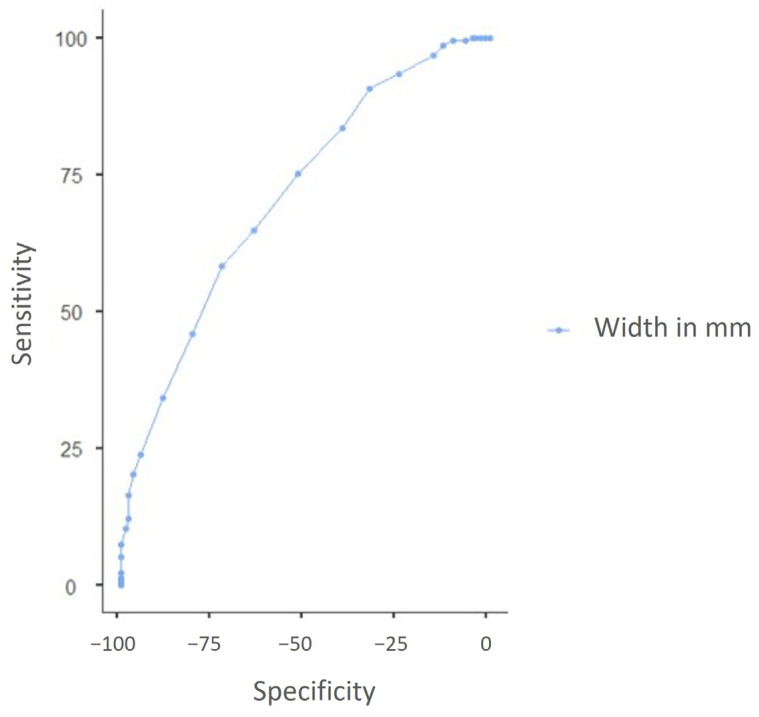
ROC curve for the width dimension of the maxillary sinus.

**Figure 5:**
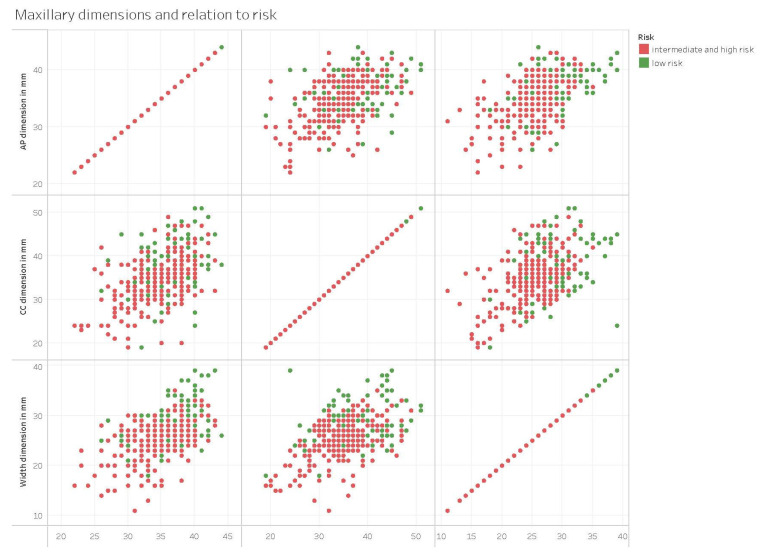
Maxillary dimensions and relation to risk. Scatter plot diagrams comparing AP, height (CC), and width dimensions of the maxillary sinus in intermediate/high-risk (red) versus low-risk (green) classes. The width dimension shows a significant visual distinction between intermediate/high and low-risk classes, indicating its potential as a critical factor in risk assessment.

Risk stratification divided the above classes into three categories based on potential exposure of the medial orbital wall during MMA: low-risk (class II), intermediate-risk (class I), and high-risk (class III). In the context of risk analysis, the intermediate and high-risk classes (class I and III) were merged, resulting in a total of 213 cases (58.7%). The remaining cases (class II) were classified as low-risk, with a total of 150 cases (41.3%). A statistically significant correlation exists between all dimensions of the maxillary sinus, the presence of a paradoxical middle turbinate, and the presence of Haller’s cells with the intermediate/high-risk class. The logistic regression model evaluated the categorization of risk (intermediate/high-risk versus low-risk). Initial investigation suggested an association between paradoxical middle turbinate and Haller’s cells; however, this association lost its statistical significance when including the width of the maxillary sinus. The width of the maxillary sinus exhibited a statistically significant negative association with intermediate/high risk (*p* < 0.001), indicating a protective influence as the width increases. The odds ratio for each 1 mm increase in width was 0.82 (95% CI: 0.76–0.88). The sensitivity, specificity, positive predictive value (PPV), negative predictive value (NPV), and area under the curve (AUC) of the width of the maxillary sinus are presented in Table 6. Furthermore, Figure 4 displays the receiver operating characteristic (ROC) curve for the width of the maxillary sinus. Figure 5 displays the maxillary sinus dimensions and their association with risk. 

## 4. Discussion

Preoperative CT scan imaging of the paranasal sinuses is essential for surgical planning in endoscopic sinus procedures, allowing for the identification of anatomical variations that could raise the risk of surgical complications [23]. The positional variations in the medial maxillary wall relative to the medial orbital wall are among the anatomical variations that are frequently overlooked during FESS presurgical planning. The attachment site of the inferior turbinate to the medial maxillary wall (lateral nasal wall) serves as an important surgical landmark, delineating the inferior margin of the MMA [17]. Another important surgical landmark of the superior aspect of the medial wall of the maxillary sinus is the maxillary ostium [24,25]. It has an anatomical relation to the midportion of the IOS, which is a thin, triangular bony junction of the medial and inferior orbital walls [26,27,28]. In standard sinonasal anatomy, the maxillary ostium is typically located within the same vertical alignment as the medial wall of the orbit [16]. In our study, more than half of the cases (55.4%) had the inferior margin of the planned MMA positioned within 2 mm, either medially or laterally, of the vertical line that intersects the IOS (Class I). According to prior studies, the inferior margin of the planned MMA is located within 2 mm of the LP in 60.1% to 80.5% of cases (Type I according to the Herzallah et al. classification) [17,18,19].

An inferior margin of the planned MMA medially located by more than 2 mm (Class II) relative to the IOS was found in 41.3% of our cases. Prior studies indicated that the inferior margin of the planned MMA is positioned medially by more than 2 mm relative to the LP in 3.5–21.3% of the cases (Type III according to the Herzallah et al. classification) [17,18,19]. In such cases, the LP is positioned more laterally and deeply relative to the planned MMA, necessitating surgical planning for more lateral dissections to prevent residuals and a revision of the surgery [17]. Residual ethmoid cells were found in up to 96.8% of patients who underwent FESS, with 79% of these cells located on the LP [29,30]. In a minority of our cases (3.3%), the inferior margin of the planned MMA was positioned more than 2 mm laterally to the IOS (Class III). Prior studies report that the inferior margin of the planned MMA is positioned laterally by more than 2 mm relative to the LP in 16.0%-24.9% of cases (Type II according to the Herzallah et al. classification) [17,18,19]. In these cases, the LP is more medially positioned relative to the planned MMA, which may increase its risk of injury during surgery [16,17]. The reported incidence of LP injury during FESS is relatively rare. Seredyka-Burduk et al. conducted a retrospective analysis involving 1658 patients who underwent endoscopic sinus surgery, revealing an injury rate of merely 0.3% to the LP [9]. Another study found a statistically significant difference (*p* < 0.01) in the incidence of orbital complications during FESS between normal patients (2.6%) and those with LP ingression (16.7%) [31]. Herzallah et al. assessed the positional variations in the LP and the associated risk of injury during surgery, revealing higher incidence in Types II and III (5.1% and 7.7%, respectively) compared to Type I (0.8%) [17].

The positional variations in the medial maxillary wall relative to the medial orbital wall have been observed to be influenced by the volume of the ethmoid and maxillary sinuses [16]. This was also demonstrated in our study, as Class II had statistically significantly higher maxillary sinus dimensions and volumes than other classes. In contrast, Class III had lower maxillary sinus dimensions and volumes than other classes. A study by Meyers et al. found that 10% of their cases showed medialization of the medial orbital wall in reference to the maxillary ostium due to ethmoid sinus hypoplasia. Furthermore, they reported that 4% of their cases exhibited maxillary sinus hypoplasia, with the medial wall of the sinus located laterally to the medial orbital wall [16]. The risk of injury to the LP during surgery is potentially increased in such cases [32]. Herzallah et al. discovered that eight sides (1.9%) of their cases exhibited maxillary sinus hypoplasia, all classified as Type II (medially positioned LP). None of these cases sustained injury to the LP during surgery, which was attributed to the meticulous preoperative planning carried out for this risky variation [17]. Another study reviewed 814 FESSs and detected maxillary sinus hypoplasia in 6.9% of the cases [33]. None of these cases experienced major postoperative complications. The authors found that FESS is safe for patients with maxillary sinus hypoplasia. However, careful radiological evaluation prior to surgery and knowledge of intraoperative endoscopic landmarks may be necessary to avoid serious complications. Moreover, the volume of the paranasal sinuses is not the sole factor influencing the variations in the medial maxillary wall or the medial orbital wall. Açar et al. examined the correlation between morphometric measurements of the orbit and types of LP. The mean values of orbital height and width were significantly greater in Type I compared to Type III (*p* < 0.05) [19].

It is worth mentioning that the positional variations in the medial maxillary wall and medial orbital wall might be influenced by various sinonasal diseases. Herzallah et al. investigated the variations in the LP in control and disease cohorts, revealing a significantly greater prevalence of Type III (laterally positioned LP) in the latter (5.4% vs. 13.5%; *p* = 0.002) [17]. Conversely, another study indicated that patients with larger polyps (Grades 3 or 4) exhibited significantly more medial LP (Type II) compared to those with smaller polyps (Grades 0, 1, or 2) [18]. They postulated that the growth pattern of larger polyps towards the nasal cavity and ciliary movement are responsible for the lack of lateral mass effect on the LP. Unfortunately, our study partially failed to resolve this controversy, as the proposed classification does not exhibit a statistically significant correlation with maxillary sinus disease based on the modified Lund–Mackay (Zinreich) score. According to Cho et al., adult patients with chronic rhinosinusitis had a significantly smaller maxillary sinus volume than the control group (*p* = 0.001) [34]. The smaller volume of the maxillary sinus will subsequently influence the position of the medial wall of the maxillary sinus. Furthermore, other anatomical variations in the OMC, beyond diseases, can influence the position of the medial wall of the maxillary sinus. Our study demonstrates a significant association between the presence of Haller’s cells and the proposed classification (*p* = 0.030). A previous study reported that an increased maxillary sinus volume is significantly associated with the presence of Haller’s cells, which ultimately influences the position of the medial maxillary wall [35]. They hypothesized that this association may be gender-related, as the incidence of Haller’s cells and the volume of the maxillary sinus are greater in males. Our investigation concurred with this hypothesis, as male subjects had significantly greater maxillary sinus dimensions and volumes than females.

Several factors have been identified to increase the risk of complications in endoscopic sinus surgery, including advanced disease, revision surgery, an inexperienced surgeon, and anatomical variations [36,37]. The findings from the logistic regression model in our study reveal a significant negative association between the width of the maxillary sinus and risk classification (*p* < 0.001), suggesting a protective influence as the width increases. The odds ratio of 0.82 for each 1 mm increase in maxillary sinus width is supported by a 95% confidence interval of 0.76 to 0.88. We hypothesized that a cut-off point of 29 mm for the maxillary sinus width can be designated as a safety margin for risk classification. These findings may guide clinical decisions, being especially valuable in preoperative planning, tailoring surgical approaches, and predicting patient outcomes. Maxillary sinus dimensions, including width, vary significantly depending on age, gender, sides, ethnicity, and disease conditions such as chronic rhinosinusitis and odontogenic health, which place a greater emphasis on preoperative planning [20,38,39]. Our investigation reveals a statistically significant difference in the width of the maxillary sinus regarding age and gender, but not when analyzed by sides. No previous research has directly addressed the correlation between maxillary sinus width and the risk of complications in endoscopic sinus surgery. Beyond contributing to risk stratifications, maxillary sinus dimensions were identified as clinically relevant factors influencing the surgical approach to fungal balls in between MMA and/or inferior metal approaches [40]. Other morphometric measurements of the maxillary sinus have recently been analyzed in relation to surgical risk stratification. Andrianakis et al. measured the width of the bony window anterior to the nasolacrimal duct, which is a crucial factor in the preoperative planning of the endoscopic pre-lacrimal window approach [41]. Their study identified gender-specific differences in these measurements, which could affect the feasibility of the endoscopic technique and thereby increase the risk of surgical complications. These findings underscore the importance of integrating gender-based anatomical assessments into preoperative planning to enhance surgical safety.

We acknowledged certain limitations in our study that might guide future research projects. While the proposed CT-based classification system provides a detailed radiological framework for assessing variations in the medial wall of the maxillary sinus, its clinical utility would be further strengthened by direct surgical validation. Future studies integrating preoperative CT assessments with prospective intraoperative findings and postoperative outcomes are warranted to validate the classification’s clinical utility and refine its application in guiding endoscopic sinus surgery. Such validation would ensure that the classification extends beyond radiological descriptions to inform surgical decision-making, ultimately enhancing procedural precision and patient outcomes. Establishing a cut-off point for safety in endoscopic sinus surgery and risk stratifications according to the proposed classification is complex and necessitates meticulous consideration of additional confounding factors, including the patient’s health condition and the surgeon’s expertise. A prospective multicenter research study is optimal for correlating the proposed classification with postoperative outcomes and other confounding factors. Furthermore, not all anatomical variations pertinent to the MMA and OMC were examined in relation to the proposed classification. These include hypertrophied or medially deviated uncinate processes, agger nasi cells, posterior septal deviations, maxillary sinus septations, and accessory ostia [42,43]. The reliance on a consensus-based review, though rigorous and involving multidisciplinary perspectives, precluded independent inter-rater reliability assessments and potentially introduced observer bias. Future studies should incorporate independent assessments to quantify reproducibility and further validate the objectivity of the proposed classification system.

## 5. Conclusions

Knowledge of the positional variations in the medial wall of the maxillary sinus relative to the medial orbital wall, as depicted in CT scans, is essential for appropriate preoperative planning in endoscopic sinus surgery. Our proposed modified classification system serves as a valuable tool for guidance towards the optimal endoscopic surgical approach, and it demonstrates relevance to risk stratification. The classification is significantly influenced by the height, width, AP dimension, and volume of the maxillary sinus. The width of the maxillary sinus, in particular, has a negative association with risk classification, according to the findings of the logistic regression model, for which we proposed a cut-off point width of 29 mm as a safety margin.

Implementing this classification in clinical practice enables surgeons to more accurately evaluate MMA boundaries and tailor their surgical approaches. Ultimately, this innovative classification not only helps to optimize surgical outcomes but also greatly reduces the incidence of postoperative complications, resulting in better overall patient care. This study emphasizes the need for continued exploration of anatomical variations relevant to MMA and their implications in surgical practice, paving the way for more refined and effective interventions in endoscopic sinus surgery.

## Figures and Tables

**Table 1:** Distribution of age among participants.

Age—Categorical	n	%
<20	22	12.0%
20–<30	79	43.2%
30–<40	50	27.3%
40–<50	16	8.7%
50–<60	13	7.1%
>60	3	1.6%

**Table 2:** Correlation of maxillary sinus dimensions and volume with gender and laterality.

	Gender			Laterality		
	Female Mean (SD)	Male Mean (SD)	*p*-Value	Right Mean (SD)	Left Mean (SD)	*p*-Value
Height	33.8 (5.23)	37.0 (6.08)	<0.001	35.1 (6.12)	36.0 (5.68)	0.165
Width	25.1 (3.90)	26.7 (4.77)	<0.001	26.4 (4.40)	25.6 (4.49)	0.099
AP	34.4 (3.47)	35.8 (4.14)	<0.001	34.9 (4.05)	35.4 (3.74)	0.274
Volume	15.6 (4.74)	19.0 (6.74)	<0.001	17.5 (6.45)	17.4 (5.81)	0.905

Maxillary sinus dimensions in mm and volume in cm^3^.

**Table 3:** Classification of the cases and their correlation with the laterality, average distance in mm from the proposed vertical line, and gender.

	Laterality					Gender		
	Right		Left			Female	Male	
	n (%)	Distance (mm)	n (%)	Distance (mm)	*p*-Value	n (%)	n (%)	*p*-Value
Class I	108 (53.7%)	0.9	93 (46.3%)	1.2		98 (48.8%)	103 (51.2%)	
Class II	68 (45.3%)	3.9	82 (54.7%)	4.0	0.255	65 (43.3%)	85 (56.7%)	0.571
Class III	7 (58.3%)	3.9	5 (41.7%)	3.4		5 (41.7%)	7 (58.3%)	

**Table 4:** Correlation of the classification of the medial maxillary wall with maxillary sinus dimensions and volume.

	Height Mean in mm (SD)	Width Mean in mm (SD)	AP Mean in mm (SD)	Volume Mean in cm^3^ (SD)
Class I	34.5 (5.72)	24.8 (4.09)	34.4 (4.13)	15.9 (5.57)
Class II	37.3 (6.68)	28.0 (4.04)	36.3 (3.26)	20.1 (5.95)
Class III	31.7 (6.54)	20.6 (4.03)	32.9 (3.75)	11.5 (4.15)

**Table 5:** Correlation of the classification of the medial maxillary wall with maxillary sinus opacifications and OMC.

	Maxillary Opacifications			OMC		
	Normal n (%)	Partial n (%)	Complete n (%)	Normal n (%)	Partial n (%)	Complete n (%)
Class I	44 (21.9%)	148 (73.6%)	9 (4.5%)	140 (69.7%)	37 (18.4%)	24 (11.9%)
Class II	42 (28.0%)	105 (70.0%)	3 (2.0%)	114 (76.0%)	21 (14.0%)	15 (10.0%)
Class III	1 (8.3%)	11 (91.7%)	0 (0.0%)	6 (50.0%)	4 (33.3%)	2 (16.7%)

**Table 6:** Sensitivity, specificity, PPV, NPV, and AUC of the width dimension of the maxillary sinus.

Cut-Point	Sensitivity (%)	Specificity (%)	PPV (%)	NPV (%)	AUC
25	58.22%	72.67%	75.15%	55.05%	0.711
26	64.79%	64.00%	71.88%	56.14%	0.711
27	75.12%	52.00%	68.97%	59.54%	0.711
28	83.57%	40.00%	66.42%	63.16%	0.711
29	90.61%	32.67%	65.65%	71.01%	0.711
30	93.43%	24.67%	63.78%	72.55%	0.711
31	96.71%	15.33%	61.86%	76.67%	0.711
32	98.59%	12.67%	61.58%	86.36%	0.711
32	99.53%	10.00%	61.10%	93.75%	0.711

## Data Availability

The data presented in this study are available from the corresponding author upon request. The data are not publicly available, as the Committee did not grant us permission to share the data.
